# Unravelling the dynamics of human development and economic growth on crude oil production based on ARDL and NARDL models

**DOI:** 10.1016/j.mex.2023.102404

**Published:** 2023-09-27

**Authors:** Jean Marie Stevy Sama, Flavian Emmanuel Sapnken, Inoussah Moungnutou Mfetoum, Jean Gaston Tamba

**Affiliations:** a Laboratory of Technologies and Applied Science, IUT Douala, PO Box 8698, Douala, Cameroon; b Transports and Applied Logistics Laboratory, University Institute of Technology, University of Douala, PO Box 8698, Douala, Cameroon; c Energy Insight-Tomorrow Today, PO Box 2043, Douala, Cameroon

**Keywords:** ARDL, NARDL, Crude oil, Causality, Cameroon, Dynamics of ARDL, NARDL and the multiplier effect

## Abstract

This paper estimates and establishes the causality between the Human Development Index (HDI), Gross Domestic Product (GDP), inflation and CO2 emissions on crude oil production (COP) in Cameroon from 1977 to 2019. To do so, the Augmented Dicky-Fuller and Zivot-Andrews stationarity tests, ARDL and NARDL modelling, as well as Toda-Yamamoto causality test are performed. Unlike previous studies on COP, this study incorporates the asymmetric impact (NARDL). The results indicate that CO2 emissions and GDP have a negative impact on COP in the long-run, while HDI and inflation have a positive impact in the short-run. GDP and HDI have a non-linear impact in the short run, while in the long-run inflation and CO2 emissions have a non-linear impact on COP. From these results, it is interesting to note that, in order to allow future generations to benefit from the oil windfall. The diversification of the Cameroonian economy, the control of inflation and the use of less polluting crude oil extraction technologies must be imperative.

• A step-by-step procedure of the ARDL, NARDL and causality test is provided.
• The multiplier effects of GDP, HDI, inflation and CO2 emissions on COP are simulated.
• The impact of GDP and HDI on COP is non-linear

## Graphical abstract

**Figure:**
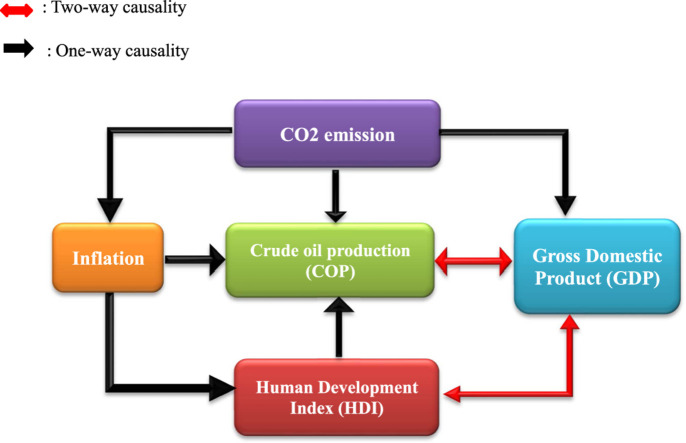

Specifications table

**Table:** 

Subject area:	Energy
More specific subject area:	Energy policy and environment
Name method:	Dynamics of ARDL, NARDL and the multiplier effect
Name and reference of original method:	S.A. Sarkodie, P.A. Owusu, How to apply the novel dynamic ARDL simulations (dynardl) and Kernel-based regularized least squares (krls), MethodsX. 7 (2020) 101,160. https://doi.org/10.1016/j.mex.2020.101160. Y. Shin, B. Yu, M. Greenwood-Nimmo, Modelling Asymmetric Cointegration and Dynamic Multipliers in a Nonlinear ARDL Framework, in: R.C. Sickles, W.C. Horrace (Eds.), Festschrift in honour of Peter Schmidt: Econometric Methods and Applications, Springer, New York, NY, 2014: pp. 281–314. https://doi.org/10.1007/978-1-4899-8008-3_9. K.P. Prabheesh, S. Kumar, A.O. Shareef, Revisiting the impact of foreign portfolio investment on stock market performance during COVID-19 pandemic uncertainty: Evidence from India, MethodsX. 10 (2023) 101,988. https://doi.org/10.1016/j.mex.2022.101988
Resource availability:	Data on CO2 emissions, inflation and GDP are from the World Bank [1] Crude oil production data are from https://www.snh.cm, and Human Development Index data are from UNDP [2].

## Introduction

The exploitation of natural resources and their use to achieve a high level of development remains a major challenge for countries [2]. As crude oil is a non-renewable and limited natural resource, it is incorporated in almost all sectors of activity [3]. Although crude oil is used in all sectors of activity, it has adverse effects on the environment, such as air pollution and greenhouse gas emissions. [4]. Thus, reconciling crude oil development with economic development and environmental protection is not an easy task for policy makers. In this respect, we assess the linear and non-linear effect of economic growth, CO2 emissions, inflation and the human development index (HDI) on crude oil production (COP). As well as causal links using ARDL, NARDL and the Toda-Yamamoto causality test. This work is in parallel with the work of Ahmad and Du [4] and the work of Danish et al. [5] and Bildirici [6] which were limited to linear ARDL modelling. This paper contributes to the literature in several ways. First, it is the first to jointly estimate the impact of CO2 emissions, HDI, inflation and economic growth on COP in Cameroon. Secondly, in addition to the ARDL model used in [4], [5], [6] which separately estimates the short- and long-run linear effects of the exogenous variables on the endogenous variables. This paper enables to captures the non-linear effects of CO2 emissions, HDI, inflation and economic growth on COP through the NARDL model which, to our knowledge, appears to have been unexplored in Central Africa and Cameroon in particular. The application of ARDL, NARDL and the Toda-Yamamoto causality test to this work allows for an efficient interpretation of the economic results. By taking into consideration the various positive or negative shocks that can be experienced in time series. Specifically, it aims to provide conclusive answers on the link between COP, economic growth, CO2 emissions and inflation in Cameroon.

## Details of the method

Before starting, it seems necessary to recall the basic notions of modelling an autoregressive delay staggered model and to highlight its qualities. An ARDL model is an autoregressive lag model capable of taking into account the temporal dimension in the explanation of a time series and making anticipations. As for the NARDL model, it is a non-linear staggered lag autoregressive model that allows capturing the different breaks or shocks that can occur on a series.

### Dataset and data sources

The data used in this paper are annual data covering the period from 1977 to 2019. The year 1977 was chosen as the starting point because it marks the first production year of crude in Cameroon. The year 2019 was chosen as the end of the study's period because of the unavailability of data for some of the variables used. Table 1 shows the data sources for the different variables used.

**Table 1:** Data description.

No of the indicator	Abbreviation	Scale of measurement	Source
Gross Domestic Product	GDP	Constant 2010 USD	WDI (2021) [7]
CO2 emissions	CO2	Kilo ton	WDI (2021) [7]
Human Development Index	HDI	Index	UNDP (2022) [8]
Crude oil production	COP	Million barrels	SNH (2022) [9]
Inflation	INF	Inflation rate	WDI (2021) [7]

### Empirical model

The theoretical conclusions demonstrating the functional relationship between COP and the variables mentioned in this study are based on the study of Reynolds and Kolodziej [10] on the relationship between COP and economic growth in the former Soviet Union. Similarly, existing and related studies have also examined the relationship between COP and other explanatory variables such as foreign investment, geopolitical risks, CO2 emissions, etc. [3,5,[11], [12], [13]]. Olanipekun and Alola [11] in particular have examined and highlighted the impact of environmental damage costs, rent and geopolitical risk on COP in the Persian Gulf. Thus, by taking into account CO2 emissions, environmental quality, human development index (HDI) and inflation (INF), this study is in line with the works carried out by Reynolds and Kolodziej [10], and Olanipekun and Alola [11]. Eq. (1) illustrates the dynamics of the links between economic growth, environmental quality and HDI on COP:

(1) $$COP_{t}=\;f\left(GDP_{t},HDI_{t},CO2_{t},\;INF_{t}\right)$$

Introducing the natural logarithm into Eq (1) makes it possible to smooth out the various variables and interpret them from the point of view of elasticities. The following empirical model is therefore used:

(2) $$LCOP_{t}=\;\beta_{0\;}+\beta_{1}LGDP_{t}+\beta_{2}LHDI_{t}+\beta_{3}LCO2_{t}+\beta_{4}\;LINF_{t}+\varepsilon_{t}$$

In Eq. (2), $LCOP_{t}$*,* $LGDP_{t}$*,* $LHDI_{t}$*,* $LCO2_{t}$ and $LINF_{t}$ are the logarithmic forms of COP, GDP, HDI, CO2 emissions and inflation respectively, while $\varepsilon_{t}$ is the error term and $\beta_{0\;}$ is a constant.

### Stationarity test

The dynamics of ARDL and NARDL modelling require that all series are level stationary (i.e. integrated of order 0, denoted I(0)) or first difference (denoted I(1)). To avoid spurious results^1^ none of the series should be integrated of order two (i.e. I(2)). In addition, the independent time series must be integrated of order, I(1) [14]. Several tests can be used to check the stationarity of the variables. For this purpose, this paper uses the Augmented Dickey-Fuller (ADF) test which is an efficient test in case of the presence of autocorrelations of the errors but very likely to reject the null hypothesis of unit root for series presenting structural breaks [15]. To overcome the shortcomings of ADF test, the Zivot-Andrews (ZA) stationarity test is used. The later takes into account different structural breaks when performing the unit root test [16]. The ADF and ZA tests are performed under the following hypothesis:

$H_{0}\;$: the series have a unit root versus

$H_{1}\;$: the series do not have a unit root.

The null hypothesis $H_{0}\;$is rejected if the p-value is less than 5 % (p-value<0.05). To control for possible spurious regression, Table 2 presents the results of the ADF and ZA stationarity tests. LCOP, LGDP, LHDI, LCO2 and LINF represent the logarithm of COP, gross domestic product (GDP), HDI, CO2 emissions and inflation respectively.

**Table 2:** Unit root test.

Value in level				Primary difference			Findings
Variables	ADF	AZ	Breakdown date	ADF	AZ	Breakdown date	
LCOP	−0.507 (0.49)	−12.84 (0.01)***	1987	−6.98 (0.00)***	-	–	I(1)
LGDP	−1.92 (0.98)	−2.147 (0.96)	1996	−0.498 (0.00)***	−6.84 (0.01)***	2002	I(1)
LINF	−3.92 (0.004)***	−5.48 (0.01)***	1993	–	–	–	I(0)
LCO2	−4.12 (0.01)***	−5.69 (0.01)***	1993	–	–	–	I(0)
LHDI	−0.019 (0.669)	−3.96 (0.167)	2007	−4.56 (0.00)***	−7.65 (0.01)***	1982	I(1)

(.) Probabilities; ***; ** and * significant at the 1 %, 5 % and 10 % level respectively							

From Table 2, it can be seen that all variables are stationary and there is no integrated series of order I(2) which meets the conditional requirements of the ARDL and NARDL model.

### Estimation of the ARDL model

Studying the relationship between COP in Cameroon and CO2 emissions, inflation, HDI and GDP is based on ARDL bounds test. This procedure is developed by Pesaran et al. [17]. It has a number of advantageous features: (i) ARDL approach provides the best linear and undistorted prediction of the long-run relationship; (ii) data size does not affect the effectiveness of the ARDL approach; (iii) the evaluation of the long-run and short-run relationships is done simultaneously; (iv) whether the series are I(0), I(1) or both, the ARDL approach can still be used [18]; (v) ARDL approach solves the problems of series endogeneity and autocorrelation; (vi) The lag sequences of the series are not always the same; and (vii) Its distinctive equation configuration makes it easier to use and interpretations are straightforward.

#### ARDL limit test

The limited ARDL test, which captures long-run and short-run effects, evaluates the following unrestricted error correction mechanism via ordinary least squares and is defined as follows

(3) $$\begin{array}{cc} \Delta LCOP_{t} & =\beta_{0}+\sum_{i=1}^{p}\beta_{1}\Delta LCOP_{t-i}+\sum_{i=0}^{p}\beta_{2}\Delta LGDP_{t-i}+\sum_{i=0}^{p}\beta_{3}\Delta LHDI_{t-i}+\sum_{i=0}^{p}\beta_{4}\Delta LINF_{t-i}\\ & \;+\sum_{i=0}^{p}\beta_{5}\Delta LCO2_{\;t-i}+\gamma_{1}LCOP_{t-i}+\gamma_{2}LGDP_{t-i}+\gamma_{3}LHDI_{t-i}+\gamma_{4}LINF_{t-i}+\gamma_{5}LCO2_{t-i}+\varepsilon_{t} \end{array}$$

Where with $\beta_{0}$ is the component of the derivative, $\varepsilon_{t}$ is the error term and assumed to be independently and normally distributed with zero mean and constant variance. The terms $\beta_{i}\;$and $\gamma_{i}$ denote the short-run and long-run multipliers respectively. Δ denotes the first difference operator, t represents the time period and p is the maximum number of lags in the model, which is determined by several criteria. The AKaike Information Criterion (AIC) is the basic criterion used in this model to determine the optimal lag. The optimal shift and model chosen are those that minimise the value of AIC.

The procedures listed below are used in this work to apply the ARDL test for cointegration limits. The first procedure is the null hypothesis which states that there is no cointegration or a long-term relationship between the series. The second is a non-null hypothesis which supports the existence of cointegration.

$H_{0}:$ ($\gamma_{i}=0;\;\forall i=1,\;2,\ldots ,\;5)$ Existence of a cointegrating relationship and,

$H_{1}:$ ($\gamma_{i}\neq 0;\;\forall i=1,\;2,\ldots ,\;5)$ Absence of a cointegrating relationship.

The cointegration of Eq. (3) is demonstrated if at least one of the long-run multipliers is different from zero. The existence of cointegration between COP and CO2 emissions, inflation, HDI and GDP is sufficiently demonstrated by rejecting the null hypothesis $H_{0}$. The second procedure is to compare the F-statistic to the two critical limits constructed by Pesaran et al. [17] in order to determine whether there are any level-related relationships. The lower critical limit is the first set of critical values, which assumes that the variables are integrated in the zero order I(0); the upper critical limit is the second set of critical values, which assumes that the variables are integrated in the one order I(1). $H_{0}$ is rejected in favour of $H_{1}$ if the F-statistic is above the upper critical limit and vice versa. If the F-statistic lies between the bounds, then the test is undecided. Thus, the calculated Fisher test statistic of value F, is compared to the critical values that form the bounds as follows:

• If Fisher > upper bound: There is cointegration;
• If Fisher < lower bound: There is no cointegration;

If lower bound < Fisher < upper bound: No conclusion

**Fig. 1:**
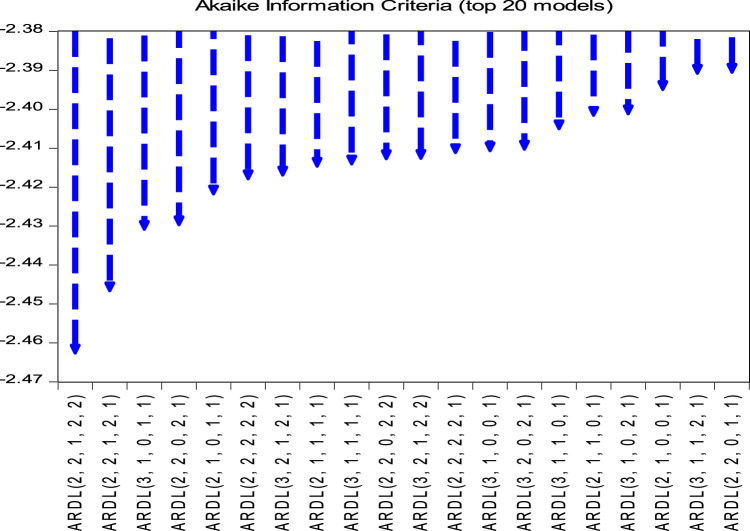
AKaike Information Criterion (AIC).

Using the AIC criterion, we can see from Fig. 1 below that the optimal shift of the series is of order 3. The ARDL model (2, 2, 1, 2, 2) is the most optimal model amongst the nineteen other models, it is the one that minimises the Akaike criterion. As for the cointegration, Table 3 presents a value of F-stat which is 7.283 higher than the values of the critical limits. The value of F-stat is higher than the values of the critical limits, we conclude that there is a cointegration relationship between COP and GDP, CO2 emissions, HDI and inflation.

**Table 3:** Cointegration test at the bounds.

Variables	COP, GDP, CO2, INF, HDI	
F-stat calculated	7.283	
Threshold	I(0)	I(1)
1 %	3.74	5.06
5 %	2.86	4.01
10 %	2.45	3.52

Having found the existence of a long-term relationship, the next step is to estimate the error correction model (ECM). The ECM can be expressed as follows:

(4) $$\Delta LCOP_{t}=\beta_{0}+\sum_{i=1}^{p}\beta_{1}\Delta LCOP_{t-i}+\sum_{i=0}^{p}\beta_{2}\Delta LGDP_{t-i}+\sum_{i=0}^{p}\beta_{3}\Delta LHDI_{t-i}+\sum_{i=0}^{p}\beta_{4}\Delta LINF_{t-i}+\sum_{i=0}^{p}\beta_{5}\Delta LCO2_{\;t-i}+\theta ECT_{t-i}+\varepsilon_{t}$$

θ is the coefficient of the error correction term$\;ECT_{t-i}$ which measures the speed of adjustment from the short-run equilibrium of the estimated ARDL model to its long-run equilibrium. The error correction coefficient must be negative and between 0 and 1 in absolute value. Tables 4 and 5 below show the short- and long-term results of the ARDL model (2,2,1,2,2) respectively. Table 4 shows a highly significant adjustment coefficient of less than 1 in absolute value.

**Table 4:** Short-term dynamics.

Variables	Coefficient	Std. Error	t-Statistic	Prob
C	23.08258	3.574537	6.457503	0.0000***
D(LGDP)	0.597528	0.289539	2.063725	0.0488**
D(LGDP (−1))	0.637666	0.637666	2.287297	0.0302**
D(LINF)	0.095756	0.173832	0.550855	0.5863
D(LCO2)	−0.010078	0.031505	−0.319896	0.7515
D(LHDI)	−0.463824	0.369625	−1.254851	0.2203
ECT (−1)	−0.276112	0.042703	−6.465918	0.0000***

***; ** and * respectively significant at the 1 % and 5 % level

**Table 5:** Long-term dynamics.

Variables	Coefficient	Std. Error	t-Statistic	Probably.
LGDP	−3.279332	0.601344	−5.453338	0.0000***
LINF	1.528338	0.420002	3.638881	0.0011***
LCO2	−0.531702	0.196522	−2.705559	0.0117**
LHDI	5.912228	1.060984	5.572403	0.0000***

With the limited ARDL test in place, as well as the short- and long-term relationships, it is necessary to check some assumptions for the viability of the results hence the next step.

#### Robustness test of the ARDL model

The different assumptions of independence, normal distribution with zero mean and constant variance made on the random disturbance terms of Eqs. (3) and (4) require to check whether it holds or not.

Therefore, serial independence is tested with the Breusch-Godfrey serial correlation LM test under the following assumptions:

$H_{0}\;$: there is no autocorrelation (prob−F>5%), against

$H_{1}\;$: There is presence of autocorrelation (prob−F<5%)*.*

Normality is tested with the Jarque-Bera test under the following assumptions:

$H_{0}\;$: the errors are normally distributed (prob−F>5%)against

$H_{1}\;$: the errors are not normally distributed (prob−F<5%).

Finally, the Breusch-Pagan-Godfrey test is used to test for zero conditional mean and constant variance. The Breusch-Pagan-Godfrey test is performed under the hypothesis:

$H_{0}\;$: There is no heteroscedasticity (prob−F>5%)against

$H_{1}\;$: There is heteroscedasticity (prob−F<5%)

When using cointegration methods, it is essential to ensure that the model is accurate, as any incorrect specification leads to instability. In order to check for parameter instability and misspecification, this study uses the cumulative sum (CUSUM) of recursive residuals and the CUSUM of squares (CUSUMSQ), as recommended by Pesaran and Pesaran [19].

**Fig. 2:**
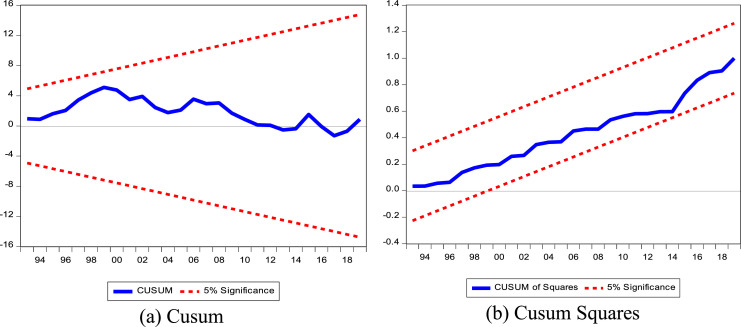
(a) Cusum and (b) Cusum Squares.

The ARDL (2,2,1,2,2) model is statistically robust. The different empirical tests presented in Table 6 confirm that the absence of heteroscedasticity, the normality of the residuals and alternatively the functional form of the specifications is correct. As for the stability of the model, Fig. 2 below shows us that the model is stable as a whole, the different Cusums do not cross the intervals defined at the 5 % threshold.

**Table 6:** Diagnostic test results of the estimated model.

Test assumptions	Tests	Values	Prob
Autocorrelation	Breusch-Godfrey	2.29	0.12
Heteroscedasticity	Breusch-Pagan-Godfrey	0.66	0.77
	Arch -Test	0.001	0.92
Normality	Jarque-Bera	3.14	0.21
Specification	Ramsey	0.22	0.64

#### Interpretation of ARDL results

Once the model has been validated, the short- and long-term results need to be interpreted. In the short term, an increase of 1 % in GDP leads to an increase in COP of 0.59 %. The time dimension is not negligible: a year ago, a 1 % increase in GDP led to a 0.63 % increase in COP. CO2 emissions, inflation and HDI are not statistically significant at the 5 % threshold in the short term. These results imply that short-term economic policies favouring wealth creation encourage Cameroon to increase its COP. On the other hand, policies related to environmental protection and human development have no impact on COP in the short term.

In the long term, a 1 % increase in CO2 emissions leads to a 0.53 % and 3.27 % decrease in COP and GDP respectively. A 1 % increase in HDI and inflation leads to an increase in COP of 5.91 % and 1.52 % respectively. These results imply that long-term economic policy initiatives to promote wealth creation will depend less and less on COP in Cameroon. As for CO2 emissions, the results imply that environmental protection policies may weaken COP. On the other hand, the Cameroonian government's long-term policy in favour of human development will stimulate COP. What would happen in the event of a sudden change (positive or negative shock) in inflation, CO2 emissions, gross domestic product and the human development index on COP? Hence the NARDL modelling.

### NARDL model procedure

The ARDL model captures the short- and long-term linear effects of the series under study (the symmetric relationship). However, it fails to capture the asymmetric relationship between the series under study (the effects of a sudden change in the variables). Moreover, the NARDL model allows for the simultaneous estimation of short- and long-term asymmetries. This explains the choice of using the non-linear ARDL model in our study. NARDL modelling does not require all variables to be integrated in the same order. Drawing on the methodology of Shin et al. [20]. The NARDL model is written as follows:

(5) $$\;LCOP_{t}=\beta_{0}+\beta_{1}^{-}LGDP_{t}^{-}+\;\beta_{2}^{+}LGDP_{t}^{+}+\;\beta_{3}^{+}LHDI_{t}^{+}+\beta_{4}^{-}LHDI_{t}^{-}+\beta_{5}^{+}LINF_{t}^{+}+\beta_{6}^{-}LINF_{t}^{-}+\;\beta_{7}^{+}LCO2_{t}^{+}+\beta_{8}^{-}LCO2_{t}^{-}+\varepsilon_{t}^{\prime }$$

Where$\;LPIB_{t}^{+}$ and $LPIB_{t}^{-}$ are the positive and negative partial sums of PIB of Eq. (6); $LCO2_{t}^{+}$ and $LCO2_{t}^{-}$ are the positive and negative partial sums of the carbon dioxide emissions CO2 emissions from Eq. (7); $LHDI_{t}^{+}$ and$\;LHDI_{t}^{-}$ represent the positive and negative partial sums of the HDI of Eq. (8); and $LINF_{t}^{+}$ and $LINF_{t}^{-}$ represent the positive and negative partial sums of inflation in Eq. (9).

(6) $$LGDP_{t}^{+}=\sum_{j=1}^{p}\Delta LPGDP_{j}^{+}=\sum_{j=1}^{p}\max\left(\Delta LGDPj,\;0\right),LGDP_{t}^{-}=\sum_{j=1}^{p}\Delta LGDP_{j}^{-}=\sum_{j=1}^{p}\min\left(\Delta LGDP_{j},\;0\right)$$

Where $\Delta LGDP_{j}^{+}$ and $\Delta LGDP_{j}^{-}$ is the process that captures increases and decreases in GDP.

(7) $$LCO2_{t}^{+}=\sum_{j=1}^{p}\Delta LCO2_{j}^{+}=\sum_{i=1}^{p}\max\left(\Delta LCO2_{j},\;0\right),\;LCO2_{t}^{-}=\sum_{j=1}^{p}\Delta LCO2_{j}^{+}=\sum_{j=1}^{p}\min\left(\Delta LCO2j,\;0\right)$$

$\Delta LCO2_{j}^{+}$ and$\;\Delta LCO2_{j}^{+}$ is a process that captures increases and decreases in CO2 emissions.

(8) $$LHDI_{t}^{+}=\sum_{j=1}^{p}\Delta LHDI_{j}^{+}=\sum_{i=1}^{p}\max\left(\Delta LHDI_{j},\;0\right),\;LHDI_{t}^{-}=\sum_{j=1}^{p}\Delta LHDI_{j}^{-}=\sum_{J=1}^{p}\min\left(\Delta LHDI_{J},\;0\right)$$

Likewise, $\Delta LHDI_{j}^{+}$ *and* $\Delta LHDI_{j}^{-}$ also capture increases and decreases in the HDI.

(9) $$LINF_{t}^{+}=\sum_{j=1}^{p}\Delta LINF_{j}^{+}=\sum_{j=1}^{p}max\left(\Delta LINF_{j},\;0\right),\;LINF_{t}^{-}=\sum_{j=1}^{p}\Delta LINF_{j}^{-}=\sum_{j=1}^{p}min\left(\Delta LINF_{J},\;0\right)\;$$

$\Delta LINF_{j}^{+}$ and $\Delta LINF_{j}^{-}$ process that captures increases and decreases in inflation. Or p is defined as the optimal lag.

#### NARDL limit test

NARDL, just like the ARDL model requires an examination of the existence of a long-run relationship between the series. Thus, NARDL representation that captures the asymmetric effects that GDP, inflation, HDI and CO2 emissions can have on COP. Consistent with the demonstration of Shin et al. [20] Eq. (10) is rewritten as follows:

(10) $$\begin{array}{cc} \Delta LCOP_{t} & =\beta_{0}+\sum_{i=1}^{p}\beta_{1}\Delta LCOP_{t-i}+\sum_{i=0}^{p}\left(\beta_{2}^{+}\Delta LGDP_{t-i}^{+}+\beta_{3}^{-}\Delta LGDP_{t-i}^{-}\right)+\sum_{i=0}^{p}\left(\beta_{4}^{+}\Delta LHDI_{t-i}^{+}+\beta_{5}^{-}\Delta LHDI_{t-i}^{-}\right)\\ & \;+\sum_{i=0}^{p}\left(\beta_{6}^{+}\Delta LINF_{t-i}^{+}+\beta_{7}^{-}\Delta LINF_{t-i}^{-}\right)+\sum_{i=0}^{p}\left(\beta_{8}^{+}\Delta LCO2_{t-i}^{+}+\beta_{9}^{-}\Delta LCO2_{t-i}^{-}\right)\\ \Delta LCOP_{t} & =\beta_{0}+\sum_{i=1}^{p}\beta_{1}\Delta LCOP_{t-i}+\sum_{i=0}^{p}\left(\beta_{2}^{+}\Delta LGDP_{t-i}^{+}+\beta_{3}^{-}\Delta LGDP_{t-i}^{-}\right)+\sum_{i=0}^{p}\left(\beta_{4}^{+}\Delta LHDI_{t-i}^{+}+\beta_{5}^{-}\Delta LHDI_{t-i}^{-}\right)\\ & \;+\sum_{i=0}^{p}\left(\beta_{6}^{+}\Delta LINF_{t-i}^{+}+\beta_{7}^{-}\Delta LINF_{t-i}^{-}\right)+\sum_{i=0}^{p}\left(\beta_{8}^{+}\Delta LCO2_{t-i}^{+}+\beta_{9}^{-}\;LCO2_{t-i}^{-}\right) \end{array}$$

Where $\sum_{i=1}^{p}\beta_{i}^{+}et\;\sum_{i=1}^{p}{\beta^{\prime }}_{i}$ captures the positive and negative short-term effects respectively. While, $\gamma_{i}^{-}$ an*d* $\gamma_{i}^{+}$ capture the long-run negative and positive effects of GDP, HDI, inflation and CO2 emissions on COP respectively. $\varepsilon_{t}^{\prime }$ is the error term that is independently and normally distributed with zero mean and constant variance. Δ denotes the first difference operator, t represents the time period and p is the maximum number of lags of the model which is determined through several criteria. The main criterion used in this model to determine the ideal lag is the AIC criterion. The optimal lag and model are those that minimise the AIC value. The ARDL test for cointegration limits is applied in this work using the procedures listed below. The first step is to reject the null hypothesis, which states that there is no cointegration or long-term relationship between the series. The second is a non-null hypothesis that confirms the existence of cointegration.

Cointegration is conducted under the following hypothesis:

$H_{0}^{\prime }:$ ($\gamma_{i}^{-}=\gamma_{i}^{+}=0;\;\forall i=1,\;2,\ldots ,\;5)$ Existence of a cointegrating relationship and,

$H_{1}^{{}^{\prime }}:$ ($\gamma_{i}^{+}\neq \gamma_{i}^{-}\neq 0;\;\forall i=1,\;2,\ldots ,\;5)$ Absence of a cointegrating relationship.

The cointegration of Eq. (10) is demonstrated if at least one of the long-run multipliers is different from zero. The existence of cointegration between COP and the various positive and negative shocks to CO2 emissions, inflation, HDI and GDP is sufficiently demonstrated by rejecting the null hypothesis $H_{0}.$ The second step is to compare the F-statistic to the two critical bounds produced by Pesaran et al. [17] to see if there are any level related relationships. The lower critical limit is the first set of critical values, which assumes that the variables are integrated at zero order I(0); the upper critical limit is the second set of critical values, which assumes that the variables are integrated at order one I(1). $H_{0}^{\prime }\;$is rejected in favour of $H_{1}^{\prime }$ if the F-statistic is above the upper critical limit and vice versa. If the F-statistic lies between the bounds, then the test is undecided. Thus, the calculated Fisher test statistic of value F, is compared to the critical values that form the bounds as follows:

• If Fisher > upper bound: Cointegration exists;
• If Fisher < lower bound: Cointegration does not exist;

If lower bound < Fisher < upper bound: No conclusion

**Fig. 3:**
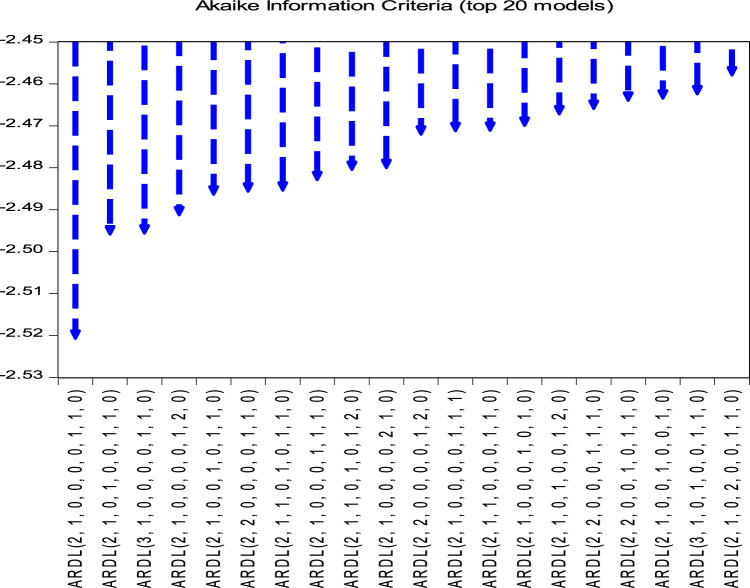
Graphical values of AIC.

The optimal lag of the series, according to the AIC criterion, is of order 3, as shown in Fig. 3. The NARDL model (2, 1, 0, 0, 0, 0, 1, 1,0) is the most optimal model amongst the nineteen other models, and it is the one that minimises the AIC criterion. As for the cointegration, Table 7 presents a value of F-stat which is 4.11 higher than the values of the critical limits. The value of F-stat being higher than the values of the critical limits, we conclude that there is a cointegration relationship.

**Table 7:** Cointegration test.

Variables	LCOP; LGDP; LCO2; LINF; LHDI	
F-stat	4.11	
Threshold	I(0)	I(1)
1 %	2.79	4.10
5 %	2.22	3.39
10 %	1.95	3.06

Once a long-term relationship has been established, the next step is to estimate the error correction model (ECM). The ECM can be expressed as follows:

(11) $$\begin{array}{cc} \Delta LCOP_{t} & =\beta_{0}+\sum_{i=1}^{p}\beta_{1}\Delta LCOP_{t-i}+\sum_{i=0}^{p}\left(\beta_{2}^{+}\Delta LGDP_{t-i}^{+}+\beta_{3}^{-}\Delta LGDP_{t-i}^{-}\right)+\sum_{i=0}^{p}\left(\beta_{4}^{+}\Delta LHDI_{t-i}^{+}+\beta_{5}^{-}\Delta LHDI_{t-i}^{-}\right)\\ & \;+\sum_{i=0}^{p}\left(\beta_{6}^{+}\Delta LINF_{t-i}^{+}+\beta_{7}^{-}\Delta LINF_{t-i}^{-}\right)+\sum_{i=0}^{p}\left(\beta_{8}^{+}\Delta LCO2_{t-i}^{+}+\beta_{9}^{-}\Delta LCO2_{t-i}^{-}\right)+\theta^{\prime }EC{T^{\prime }}_{i}+{\varepsilon^{\prime }}_{i} \end{array}$$

$\theta^{\prime }$is the coefficient of the error correction term$\;ECT_{i}^{\prime }\;$which measures the speed of adjustment from the short-run equilibrium of the estimated NARDL (2, 1, 0, 0, 0, 0, 1, 1,0) model to its long-run equilibrium. The error correction coefficient must be negative and between 0 and 1 in absolute value. Tables 8 and 9 below show the short- and long-term results of the NARDL model respectively. Table 8 shows a highly significant adjustment coefficient of less than 1.

**Table 8:** Short-term dynamics NARDL.

Variable	Coefficient	Std. Error	t-Statistic	Prob
C	0.927186	0.137765	6.730188	0.0000
D(LCOP (−1))	0.475067	0.049280	9.640087	0.0000***
D(LGDP_POS)	0.774523	0.287334	2.695551	0.0119**
D(LCO2_NEG)	0.016774	0.036447	0.460241	0.6490
D(LHDI_POS)	−1.648821	0.348283	−4.734142	0.0001***
$ECT_{i}^{\prime }$	−0.255482	0.036894	−6.924696	0.0000***

*** and ** significant at the 1 % and 5 % level respectively

**Table 9:** Long-term dynamics of NARDL.

Variables	Coefficient	Std. Error	t-Statistic	Probably.
LGDP_POS	−2.397247	0.593734	−4.037575	0.0004***
LGDP_NEG	0.822433	2.849750	0.288598	0.7751
LINF_POS	0.952534	0.409318	2.327127	0.0277**
LINF_NEG	6.632144	12.21469	0.542965	0.5916
LCO2_POS	0.085377	0.260884	0.327261	0.7460
LCO2_NEG	−0.537701	0.322449	−1.667555	0.1070
LHDI_POS	2.894734	1.498184	1.932161	0.0639*
LHDI_NEG	5.032520	3.358598	1.498399	0.1456

***; ** and * significant at the 1 %, 5 % and 10 % level respectively				

As the short- and long-term relationships of the NARDL model are estimated for greater certainty, it is necessary to check the reliability of the model. The reliability of the NARDL model results in this study requires the verification of certain assumptions, hence the next step.

#### Robustness test of the NARDL model

The NARDL model (2,1,0,0,0,0,1,1,0) could be valid, if it takes into account the different assumptions of independence, normal distribution made on the error term of Eqs. (10) and (11). Thus, to check the robustness of the NARDL model, we proceed in exactly the same way as the ARDL model in section 2.4.2.

**Fig. 4:**
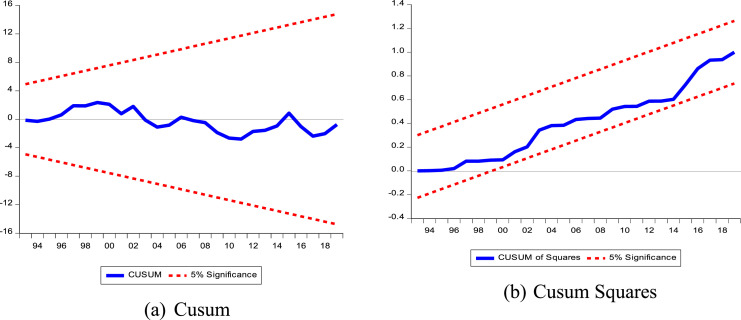
(a) Cusum and (b) Cusum Squares.

Statistically, the NARDL model (2, 1, 0, 0, 0, 0, 1, 1, 0) is valid. The different empirical tests presented in Table 10 confirm the absence of heteroscedasticity; the normality of the residuals and alternatively the functional form of the specifications is correct. In addition to this, the NARDL model (2, 1, 0, 0, 0, 0, 1, 1, 0) is globally good and explains 97 % of the dynamics of the different shocks of the different explanatory series on COP over the period 1977 to 2019. Following the assessment, a look at the stability (Fig. 4) shows that the NARDL model (2, 1, 0, 0, 0, 0, 1, 1, 0) is stable overall. This is because the individual Cusums do not cross the intervals defined at the 5 % threshold. The Wald test is then applied in the next step in order to confirm the asymmetric effect in the short and long term as shown in Tables 8 and 9.

**Table 10:** Results of the different diagnostic tests of the estimated model.

Test Hypotheses	Tests	Values	Prob
Autocorrelation	Breusch-Godfrey	3.34	0.514
Heteroscedasticity	Breusch-Pagan-Godfrey Arch-Test	0.64 0.79	0.705 0.37
Normality	Jarque-Bera	3.53	0.17
Specification	Ramsey	0.29	0.76

#### Wald test

The NARDL model (2,1,0,0,0,0,1,1,0) being robust and cointegration being justified. To confirm the asymmetric short-run and long-run effects of GDP, CO2 emissions, HDI and inflation on COP in Cameroon. The standard Wald test is used and is based on the following assumptions:

• Short-term assumption :

$H_{0}:$ ($\beta_{i}^{-}=\beta_{i}^{+}=\beta =0;\;\forall i=1,\;2,\ldots ,\;5)$ absence of asymmetry and,

$H_{1}:$ ($\beta_{i}^{-}\neq \beta_{i}^{+}\neq \beta \neq 0\;;\;\forall i=1,\;2,\ldots ,\;5)$ presence asymmetry.

• Long-term assumption

$H_{0}:$ ($\gamma_{i}^{-}=\gamma_{i}^{+}=\gamma =0;\;\forall i=1,\;2,\ldots ,\;5)$ absence of asymmetry and,

$H_{1}:$ ($\gamma_{i}^{+}\neq \gamma_{i}^{-}\neq \gamma \;\neq 0;\;\forall i=1,\;2,\ldots ,\;5)$ presence asymmetry.

The symmetrical short- or long-term effects of GDP, HDI, CO2 emissions and inflation are confirmed by the rejection of the null hypothesis $H_{0}.$ The hypothesis $H_{0}\;$is rejected if the calculated probability is greater than prob-F>5%. The results of the Wald test presented in Table 11 show that GDP and HDI have asymmetric effects in the short run. The results of the Wald test in Table 11 show that GDP and HDI have asymmetric effects in the short run, while in the long run CO2 emissions and inflation have asymmetric effects.

**Table 11:** Wald test.

Variables	Short term		Long term	
	F-stat	Prob	F-stat	Prob
LGDP	6.27	0.016**	1.324	0.256
LHDI	11.50	0.0017***	0.203	0.654
LCO2	1.206	0.2794	13.0	0.00***
LINF	–	–	26.89	0.00***

*** and ** significant at the 1 % and 5 % levels respectively				

#### Interpretation of NARDL results

Short-term asymmetric analysis indicates strong non-linearity between HDI and GDP. A positive shock in GDP of 1 % leads to an increase in COP of 0.77 %. A negative shock in HDI of 1 % leads to a decrease in COP of 1.64 %. This means that in the event of a fall in HDI, possibly due to education, the resulting effect will be a significant fall in COP. The positive GDP shock implies that a radical change in economic policy aimed at accelerating wealth creation in Cameroon would lead to a significant increase in COP.

In the long term, asymmetric analysis indicates a strong non-linearity between inflation and GDP on COP. Thus, a positive shock of 1 % in inflation, as shown in Table 9, leads to an increase in COP of 0.95 %. This means that a sudden rise in the prices of goods and services would lead to an increase in COP. CO2 emissions are not significant. This implies that oil production could take place independently of a positive or negative shock to CO2 emissions. The Wald test confirms the asymmetric short- and long-term effects of GDP, HDI, CO2 emissions and inflation on COP.

#### Dynamics of the multiplier effect

The dynamic multiplier effect is evaluated, or a variation of 1 %, 5 % or 10 %. ${\text{LGDP}}_{t}^{+},$ ${\text{LGDP}}_{t}^{-}$, $\text{LCO}2_{t}^{+},\text{LCO}2_{t}^{-},{\text{LHDI}}_{t}^{+},{\text{LHDI}}_{t}^{-}$, ${\text{LINF}}_{t}^{+}$ and ${\text{LINF}}_{t}^{-}$ can be derived as follows:

(12a) $$H_{m}^{-}=\sum_{i=0}^{m}\frac{\partial COP_{t+j}}{\partial LGDP_{t}^{-}}\;H_{m}^{+}=\sum_{i=0}^{m}\frac{\partial COP_{t+j}}{\partial LGDP_{t}^{+}}$$

(12b) $$K_{m}^{-}=\sum_{i=0}^{m}\frac{\partial COP_{t+j}}{\partial LIDH_{t}^{-}},\;K_{m}^{+}=\sum_{i=0}^{m}\frac{\partial COP_{t+j}}{\partial LIDH_{t}^{+}}$$

(12c) $$D_{m}^{-}=\sum_{i=0}^{m}\frac{\partial COP_{t+j}}{\partial LINF_{t}^{-}},\;D_{m}^{+}=\sum_{i=0}^{m}\frac{\partial COP_{t+j}}{\partial LINF_{t}^{+}}$$

(12d) $$B_{m}^{-}=\sum_{i=0}^{m}\frac{\partial COP_{t+j}}{\partial LCO2_{t}^{-}},B_{m}^{+}=\sum_{i=0}^{m}\frac{\partial COP_{t+j}}{\partial LCO2_{t}^{+}}$$

From Eqs. (10) and (12a–d), the long-term asymmetric coefficient is estimated as $=-\frac{\lambda^{+}}{\gamma };\;K-=-\frac{\lambda^{-}}{\gamma }$ given that *p→∞*

**Fig. 5:**
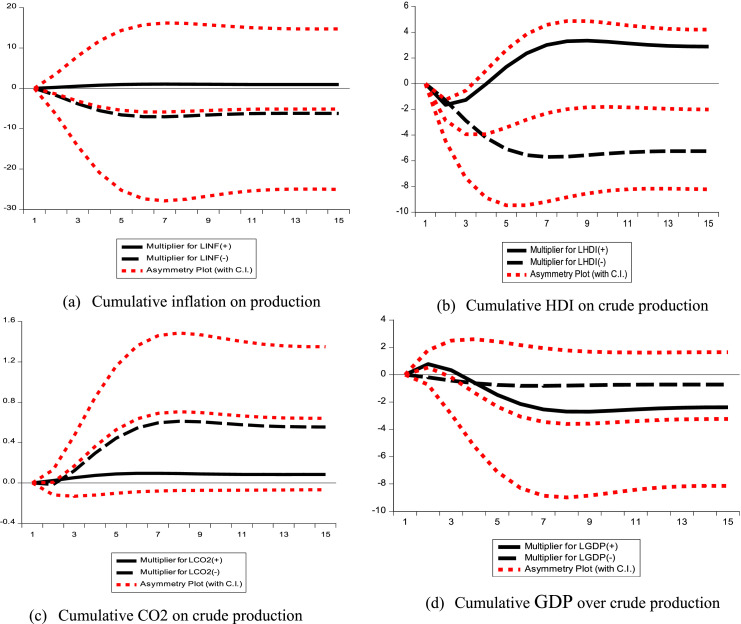
(a) Cumulative inflation; (b) Cumulative HDI; (c) Cumulative carbon dioxide and (d) Cumulative GDP.

The illustrations in Fig. 5 show that the positive GDP shock clearly dominates the negative shock in the overall asymmetry. On the other hand, the negative shock from inflation, HDI and CO2 emissions clearly dominates the positive shock in the global asymmetry.

### Toda-Yamamoto causality

Since the series are combined in different orders, the traditional Granger causality test is inoperative. This makes it possible to use the Toda and Yamamoto causality test [21]. In concrete terms, this involves estimating a level-corrected vector autoregression (VAR), which is to serve as the basis for the causality test, under the hypothesis of a probable cointegration between the series. The absence of a causal relationship between the series is specified by the null hypothesis. Thus, the causality test procedure of [21] is as follows:

• Find the maximum order of integration (dmax) of the sub-study series using conventional stationarity tests;
• Determine the optimal lag or shift (k) of the model's VAR or autoregressive polynomial (AR) using the information criteria (AIC);
• Estimating a level-augmented VAR of order ≪p=k+dmax≫.Concerning the estimation of the VAR in augmented level, the stationarity conditions of the series will define the number of lags to be added to the VAR. In fact, for stationary series in level, no lag is added to the VAR (standard test procedure); on the other hand, for I(1) series, a lag will be added to the VAR, and so on.
• Check the robustness of the VAR model (p=k+dmax) using various available diagnostic tests [22].
• The Wald test is performed on the initial parameters k parameters and provides an asymptotic Chi-square distribution with degrees of freedom (for more information, [22]).

The various causal equations are presented as follows for the sake of parsimony the list of equations is not exhaustive:

**Model 1:** COP and GDP

(13) $$\begin{array}{cc} LCOP_{t} & =\;\beta_{0}+\sum_{i=1}^{p}\beta_{1i}LCOP_{t-i}+\sum_{j=k+1}^{dmax}\beta_{1j}LCOP_{t-j}+\sum_{i=1}^{p}\alpha_{1i}LGDP_{t-i}+\sum_{j=k+1}^{dmax}\alpha_{1j}LGDP_{t-j}\;+\sum_{i=1}^{p}\mu_{1i}LHDI_{t-i}\\ & \;+\sum_{j=k+1}^{dmax}\mu_{1j}LHDI_{t-j}++\sum_{i=0}^{p}\theta_{1i}LINF_{t-i}+\sum_{j=k+1}^{dmax}\theta_{1j}LINF_{t-j}+\sum_{i=1}^{p}\phi_{1i}LCO2_{\;t-i}+\sum_{j=k+1}^{dmax}\phi_{1j}LCO2_{\;t-i}+\varepsilon_{1t} \end{array}$$

**Model 2:** GDP and COP

(14) $$\begin{array}{cc} LCOP_{t} & =\;\beta_{0}+\sum_{i=1}^{p}\beta_{1i}LCOP_{t-i}+\sum_{j=k+1}^{dmax}\beta_{1j}LCOP_{t-j}+\sum_{i=1}^{p}\alpha_{1i}LGDP_{t-i}+\sum_{j=k+1}^{dmax}\alpha_{1j}LGDP_{t-j}\;+\sum_{i=1}^{p}\mu_{1i}LHDI_{t-i}\\ & \;+\sum_{j=k+1}^{dmax}\mu_{1j}LHDI_{t-j}++\sum_{i=0}^{p}\theta_{1i}LINF_{t-i}+\sum_{j=k+1}^{dmax}\theta_{1j}LINF_{t-j}+\sum_{i=1}^{p}\phi_{1i}LCO2_{\;t-i}+\sum_{j=k+1}^{dmax}\phi_{1j}LCO2_{\;t-i}+\varepsilon_{1t} \end{array}$$

COP causes GDP if $\alpha_{1i}\neq 0,\;\forall i=1,\;2,\ldots ,\;k$ in Eq. (11). Conversely, the GDP causes the production of crude oil if,$\;\forall i=1,\;2,\ldots ,\;k,\beta_{1i}\neq 0$ in Eq. (12). There is a two-way causality between COP and GDP if $\alpha_{1i}\neq 0$ and$\;\beta_{1i}\neq 0,\;\forall i=1,\;2,\ldots ,\;k$ in Eqs. (11) and (12). Finally, there is no causality between oil production and GDP if $\alpha_{1i}=\beta_{1i}=0,\;\forall i=1,\;2,\ldots ,\;k$ in Eqs (11) and (12).

**Model 3:** HDI and COP

(15) $$\begin{array}{cc} LHDI_{t} & =\;\beta_{0}+\sum_{i=1}^{p}\beta_{1i}LCOP_{t-i}+\sum_{j=k+1}^{dmax}\beta_{1j}LCOP_{t-j}+\sum_{i=1}^{p}\alpha_{1i}LGDP_{t-i}+\sum_{j=k+1}^{dmax}\alpha_{1j}LGDP_{t-j}\;+\sum_{i=1}^{p}\mu_{1i}LHDI_{t-i}\\ & \;+\sum_{j=k+1}^{dmax}\mu_{1j}LHDI_{t-j}++\sum_{i=0}^{p}\theta_{1i}LINF_{t-i}+\sum_{j=k+1}^{dmax}\theta_{1j}LINF_{t-j}+\sum_{i=1}^{p}\phi_{1i}LCO2_{\;t-i}+\sum_{j=k+1}^{dmax}\phi_{1j}LCO2_{\;t-i}+\varepsilon_{1t} \end{array}$$

**Model 4:** Inflation and COP

(16) $$\begin{array}{cc} LINF_{t} & =\;\beta_{0}+\sum_{i=1}^{p}\beta_{1i}LCOP_{t-i}+\sum_{j=k+1}^{dmax}\beta_{1j}LCOP_{t-j}+\sum_{i=1}^{p}\alpha_{1i}LGDP_{t-i}+\sum_{j=k+1}^{dmax}\alpha_{1j}LGDP_{t-j}\;+\sum_{i=1}^{p}\mu_{1i}LHDI_{t-i}\\ & \;+\sum_{j=k+1}^{dmax}\mu_{1j}LHDI_{t-j}++\sum_{i=0}^{p}\theta_{1i}LINF_{t-i}+\sum_{j=k+1}^{dmax}\theta_{1j}LINF_{t-j}+\sum_{i=1}^{p}\Phi_{1i}LCO2_{\;t-i}+\sum_{j=k+1}^{dmax}\Phi_{1j}LCO2_{\;t-i}+\varepsilon_{1t} \end{array}$$

**Model 5:** Carbon dioxide emissions and COP

(17) $$\begin{array}{cc} LCO2_{t} & =\;\beta_{0}+\sum_{i=1}^{p}\beta_{1i}LCOP_{t-i}+\sum_{j=k+1}^{dmax}\beta_{1j}LCOP_{t-j}+\sum_{i=1}^{p}\alpha_{1i}LGDP_{t-i}+\sum_{j=k+1}^{dmax}\alpha_{1j}LGDP_{t-j}\;+\sum_{i=1}^{p}\mu_{1i}LHDI_{t-i}\\ & \;+\sum_{j=k+1}^{dmax}\mu_{1j}LHDI_{t-j}++\sum_{i=0}^{p}\theta_{1i}LINF_{t-i}+\sum_{j=k+1}^{dmax}\theta_{1j}LINF_{t-j}+\sum_{i=1}^{p}\Phi_{1i}LCO2_{\;t-i}+\sum_{j=k+1}^{dmax}\Phi_{1j}LCO2_{\;t-i}+\varepsilon_{1t} \end{array}$$

**Fig. 6:**
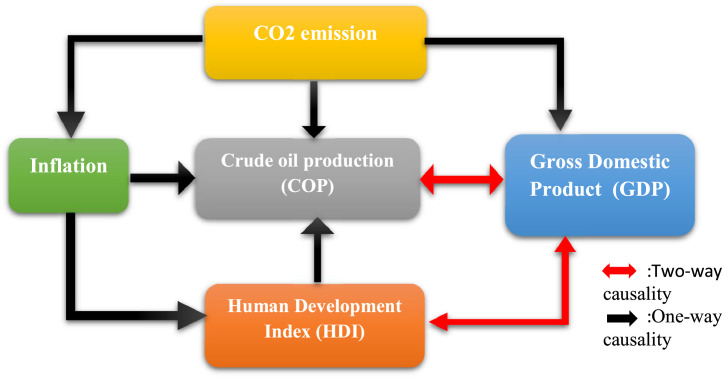
Causal links between the series.

The interpretation of the causal inference of models 3 to 5 follows the same logic as for models 1 and 2. Before performing the Toda-Yamamoto test, the stationarity of the series should be checked. Table 2 presents the results of the stationarity of the series. The finding is that the series are integrated at level and first difference, the maximum integration order is set to one I(1). The optimal lag length using the AIC criterion is set to 3, which allows the VAR to satisfy both stability conditions and the absence of autocorrelations. The Toda-Yamamoto causality obtained from the VAR is presented in Table 12. Fig. 6 summarises the causal links between the different variables.

**Table 12:** Causality between series.

Variables	Explanatory variables				
dependant	lcop	lgdp	linf	lco2	lhdi
LCOP	–	32.65*** (0.00)	14.86*** (0.00)	5.64* (0.06)	32.23*** (0.00)
LGDP	6.84** (0.03)	–	2.46 (0.29)	21.03*** (0.00)	17.16*** (0.00)
LINF	2.78 (0.25)	2 (0.36)	–	7.39** (0.02)	2.55 (0.27)
LCO2	0.83 (0.65)	1.31 (0.51)	2.26 (0.32)	–	1.75 (0.41)
LHDI	4.57 (0.102)	15.54*** (0.00)	12.68*** (0.00)	0.67 (0.73)	–

(.) Probabilities; ***; ** and* respectively at the 1 %; 5 % and 10 % significant level

## Conclusion

ARDL and NARDL models and the Toda-Yamamoto causality test were used in this study to estimate and establish the causal links between GDP, HDI, CO2 emissions and inflation on COP in Cameroon over the period 1977 to 2019. The combination of the ARDL and NARDL method and the Toda-Yamamoto causality test provides sufficient information between the variables to better guide policy makers in their decision making. Inflation control should be an essential element of economic policy, as it has a positive impact on COP. Diversification of the Cameroonian economy should also be an essential element for the country's development. A positive shock to economic growth has a negative impact on COP. Finally, the rents from COP should be directed towards education, health and, in short, towards social projects to improve the living conditions of the population. There is no causality between oil production and the HDI. Environmental regulations could be further strengthened to ensure that COP has less impact on the environment. This study does not include institutional quality as a series that can explain COP. However, institutional quality is very important in the decision-making process of oil resource investments. It would be interesting for future research to examine the relationship between oil production and institutional quality.

## Funding

This research did not receive any specific grant from funding agencies in the public, commercial, or not-for-profit sectors.

## CRediT authorship contribution statement

**Jean Marie Stevy Sama:** Conceptualization, Methodology, Software, Writing – original draft. **Flavian Emmanuel Sapnken:** Validation, Data curation, Visualization, Investigation. **Inoussah Moungnutou Mfetoum:** Software, Writing – review & editing. **Jean Gaston Tamba:** Supervision, Validation, Writing – review & editing.

## Declaration of Competing Interest

The authors declare that they have no known competing financial interests or personal relationships that could have appeared to influence the work reported in this paper.

## Data Availability

Data will be made available on request.

## Footnotes

1 A situation that suggests an optimistic outcome when this is not the case (false results).
